# Evaluation of Probenecid Against Filovirus Replication in Vero E6 Cells

**DOI:** 10.3390/v18010043

**Published:** 2025-12-26

**Authors:** Kendra Alfson, Ricardo Carrion, Ralph A. Tripp, Chris Cirimotich, David E. Martin

**Affiliations:** 1 Texas Biomedical Research Institute, San Antonio, TX 78227, USA; kalfson@txbiomed.org (K.A.); ricardo.carrionjr@wisc.edu (R.C.J.); 2 Department of Infectious Diseases, University of Georgia, Athens, GA 30602, USA; ratripp@uga.edu; 3 Battelle Biomedical Research Center, West Jefferson, OH 43162, USA; cirimotichc@battelle.org; 4 TrippBio, Inc., Jacksonville, FL 32256, USA

**Keywords:** probenecid, Ebolaviruses, EBOV, SUDV, MARV, Vero E6 cells

## Abstract

In human and non-human primates, filoviruses, e.g., Ebolaviruses, cause severe hemorrhagic fever for which there are few therapeutic options. While there are licensed vaccines and therapeutics for Ebola virus disease, there is no approved vaccine or treatment for other Ebola diseases. There is a need for broad-spectrum antivirals to treat Ebola virus (EBOV), Sudan virus (SUDV), and Marburg virus (MARV). We have previously demonstrated that probenecid, an FDA-approved drug with a safety profile spanning over 7 decades, is safe and effective in preventing the replication of influenza A viruses, SARS-CoV-2, and other RNA respiratory viruses, such as HMPV and RSV, both in vitro and in vivo. In this study, probenecid was shown to inhibit the replication of infectious EBOV, SUDV, and MARV in Vero E6 cells, with IC50 Values of 3 μM, 8 μM, and 13 μM, respectively. It also reduced plaque size in infected Vero cell lawns, suggesting reduced virus spread. These studies show that probenecid is an effective, broad-spectrum, host-directed antiviral drug.

## 1. Introduction

Filoviruses in the genus Orthoebolavirus (i.e., Ebola virus, EBOV; Sudan virus, SUDV) and Orthomarburgvirus (e.g., Marburg virus, MARV) are enveloped, negative-sense RNA viruses that cause severe viral hemorrhagic fever in humans and nonhuman primates (NHPs), with case fatality rates that can approach 50–90% depending on the virus species, outbreak setting, and access to supportive care [1,2]. Disease often begins as an acute febrile illness with gastrointestinal symptoms and rash, and may rapidly progress to vascular leak, coagulopathy, shock, and multi-organ failure within 7–10 days of exposure. Transmission occurs through direct contact with the blood or other body fluids of infected individuals or animals, with an elevated risk in healthcare and funerary settings, particularly in the absence of stringent infection prevention and control practices. Experimental and NHP studies indicate that low virus inocula can be sufficient to establish infection and lead to severe disease, underscoring the high-risk nature of exposure and the need for layered countermeasures [3,4].

Filoviruses are zoonotic, with bats serving as natural reservoirs. Spillover into humans may occur through direct or indirect contact with infected wildlife, followed by sustained human-to-human transmission [1,2]. Recurrent outbreaks of EBOV, SUDV, and MARV in sub-Saharan Africa highlight both the ecological persistence of these pathogens and the ongoing threat to global health security. The outbreak response includes detection and infection-control practices, such as contact tracing and proper burial, and may also include ring vaccination to interrupt transmission. Monoclonal antibody (mAb) therapies, such as Ebanga, are FDA-approved treatments for Ebola disease, specifically Zaire ebolavirus, that block viral entry into cells by targeting the virus’s surface protein [5]. Other therapeutic mAbs, such as REGN-EB3 and mAb114, have also been recommended for use in patients with the disease by the WHO [6]. These therapies offer immediate, passive immunity, helping vulnerable populations who cannot mount an effective response to a vaccine, and can be used to treat the infection. There are currently no approved vaccines or therapeutics for SUDV or MARV, leaving significant gaps in preparedness for non-Zaire filoviruses. This disparity supports the development of broadly active, deployable antivirals that can complement existing measures across filovirus species.

Host-directed antivirals can provide broad coverage against divergent viruses. They may be less susceptible to loss of efficacy due to viral genetic drift and drug-induced resistance. Probenecid is a safe and FDA-approved small molecule with more than seven decades of clinical use, historically as a uricosuric agent and to modulate renal tubular secretion of β-lactam antibiotics. Probenecid treatment modulates membrane channels and transporters and alters host signaling pathways implicated in virus–host interactions and inflammation [7,8,9,10,11,12,13,14,15]. Previous studies have shown probenecid’s antiviral activity against diverse RNA respiratory viruses, including influenza A viruses, SARS-CoV-2, respiratory syncytial virus (RSV), and human metapneumovirus (HMPV), with both in vitro and in vivo efficacy [8,9,10,11,12,13,14,16]. Probenecid has been shown to inhibit mitogen-activated protein kinase (MAPK) pathways, e.g., extracellular signal-regulated kinase (ERK) and c-Jun N-terminal kinase (JNK), as well as NLRP3 inflammasome activity, suggesting the potential to reduce both viral replication and virus-induced inflammatory responses [8,9,10,15]. Given the established human safety profile and broad antiviral activity, we evaluated whether probenecid inhibits the replication of EBOV, SUDV, and MARV in Vero E6 cells within tissue culture plates at concentrations of 1000, 100, 10, 1, 0.1, 0.01, and 0 µM. The study was conducted under high-containment conditions at the Texas Biomedical Research Institute (Texas Biomed) in its BSL-4 laboratories, using a wild-type infectious virus.

## 2. Materials and Methods

### 2.1. Vero E6 Cells and Overlays

Vero E6 cells, which have an epithelial morphology and were cloned from the kidney of an African green monkey (ATCC, Manassas, VA, USA), were seeded onto 6-well plates (7–9.5 × 10^5^ cells/well), propagated in Dulbecco’s Modified Eagle Medium (DMEM; Thermofisher, Waltham, MA, USA) containing 10% heat inactivated FBS (Gibco, Grand Island, NY, USA), and allowed to adhere overnight. For evaluation, the cells were incubated with approximately 50 PFU virus for 1 h, in a 6-well plate at 37 ± 2 °C, 5% CO_2_, 95% RH. After 1 h of incubation, the inoculum was removed from the wells and replaced with 2 mL primary overlay. Overlay consisted of 2X Modified Eagle Medium (MEM) with 4% heat-inactivated FBS (Gibco, Grand Island, NY, USA) and 2mM sodium pyruvate (Gibco) and an appropriate concentration of test or control article, mixed 1:1 with agarose (Sigma, St. Louis, MO, USA) dissolved in distilled water to a final agarose concentration of 0.5%. On day 7 pi, 2 mL of the secondary overlay was added. The secondary overlay was prepared in the same manner as the primary overlay, with the addition of 4% (final concentration) neutral red and the omission of the test article. On day 8, plates were scanned for plaque enumeration.

### 2.2. Probenecid

A working stock of probenecid (CAS No.: 57-66-9, Sigma-Aldrich, St. Louis, MO, USA) was dissolved in DMSO (Sigma-Aldrich), and dilutions of the working stock were resuspended in PBS (Gibco). TrippBio, Inc. supplied the probenecid as a 100 mM frozen solution. This was tested at final concentrations of 1000, 100, 10, 1, 0.1, 0.01, and 0 µM. From the frozen stock, initial dilutions were made in infection media (Dulbecco’s Modified Eagle Medium (DMEM) supplemented with 2% FBS) and final dilutions for use were made in 2X MEM overlay media supplemented with 4% FBS (for a final concentration of 2% FBS); test or control article concentrations in the 2X MEM media were also prepared at 2X (e.g., 2000 µM to achieve the final concentrations listed above, after the 1:1 dilution with agarose). DMSO at an appropriate concentration was used as a control. All test materials and media were maintained at 37 °C throughout to prevent probenecid precipitation. Probenecid was used therapeutically only in these studies (i.e., included in overlay media only, as described above). Given the efficacy observed and the long-term intent for use in treatment settings, prophylactic treatment was not tested.

### 2.3. Viruses

A second cell-culture passage of Marburg virus (MARV; Angola variant) provenance is from a fatal human case in Uige, Angola, 2005 [17], Sudan virus (SUDV; Gulu variant); provenance was from a fatal human case in Uganda, Gulu, in 2000 [18], and Ebola virus (EBOV; Kikwit variant); provenance is from a fatal human case in Kikwit, Democratic Republic of the Congo, 1995), were obtained from Dr. Tom Ksiazek (NIAID’s World Reference Center for Emerging Viruses and Arboviruses (WRCEVA) at UTMB Health Galveston National Laboratory) and propagated at Texas Biomed. The stock viruses were passaged for a third time in Vero E6 cells under growth conditions of 37 °C, 5% CO_2_. The multiplicity of infection (MOI) was 0.001 in minimal essential medium (MEM, Gibco) supplemented with 2% fetal bovine serum (FBS). The P3 stocks of EBOV (1.2 × 10^5^ PFU/mL), MARV (10^5^ PFU/mL), and SUDV were passaged a fourth time to generate P4 stocks in Vero E6 cells at approximately 1 × 10^5^ PFU/mL. The stocks were stored at −65 °C.

### 2.4. Compliance Statement

This exploratory study is exempt from 21 CFR Part 58, Good Laboratory Practice (GLP) Regulations. All portions of this study performed at Texas Biomed adhered to the study protocol and, where applicable, Texas Biomed Standard Operating Procedures, and generally recognized good documentation practices. Filoviruses are Risk Group 4 (maximum containment) pathogens; as such, studies occurred at BSL-4. All manipulations involving live filoviruses were performed in the Centers for Disease Control and Prevention (CDC)-accredited Biosafety Level 4 (BSL-4) at Texas Biomed.

### 2.5. Statistical Analysis

The percent reduction in viral replication in vitro following probenecid treatment was determined. To achieve this, the raw virus plaque counts were averaged, and the percent reduction was calculated relative to the average PFU of the virus-only control wells (i.e., the percent reduction in PFU relative to the average PFU of the virus-only control wells). The percent reduction at each concentration was analyzed in GraphPad Prism (Version 9.1.0) using a nonlinear regression (inhibitor vs. response, variable slope) to estimate the concentration expected to yield a 50% reduction in infectivity. We visually evaluated the relative plaque size when treated with different concentrations of probenecid.

## 3. Results

**Figure 1:**
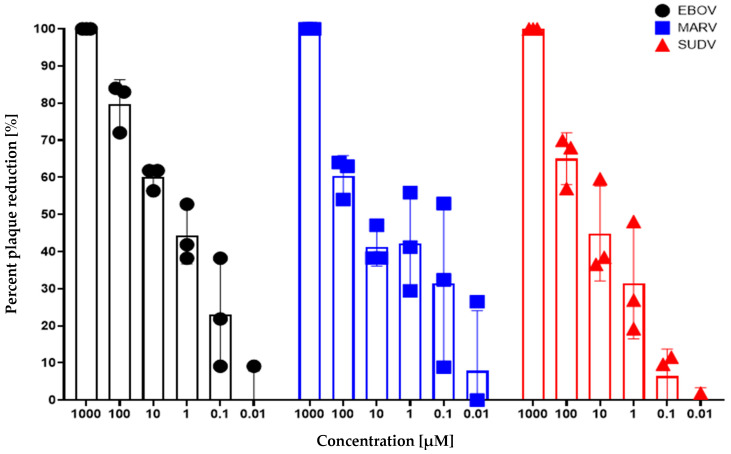
Probenecid treatment and the percent plaque reduction for filoviruses. An in vitro plaque-inhibition assay was used to determine whether probenecid inhibited replication of filoviruses (EBOV, MARV, and SUDV). The assay was performed in biological triplicate for each virus and each concentration. Plaques in each well were counted to calculate the quantity of PFU present. Plaque counts were compared for each test article concentration with wells containing the control article only to determine the percent reduction in infectivity.

Probenecid inhibited plaque formation of EBOV, MARV, and SUDV at 1000 µM (100% inhibition), 100 µM, 10 µM, and 1 µM, with the highest efficacy against EBOV (Table 1, Figure 1). Probenecid treatment at 0.1 µM was also effective in inhibiting viral replication, as evidenced by reduced plaque formation for EBOV and MARV; however, efficacy was lower than at higher probenecid concentrations. Plaque counts were comparable to those in control wells for all three viruses at the lowest concentration. The estimated in vitro IC_50_ values for probenecid against EBOV, MARV, and SUDV were 3 µM, 8 µM, and 13 µM, respectively (Table 1). The percent reduction in virus replication in vitro following probenecid treatment is shown in Figure 1.

**Figure 2:**
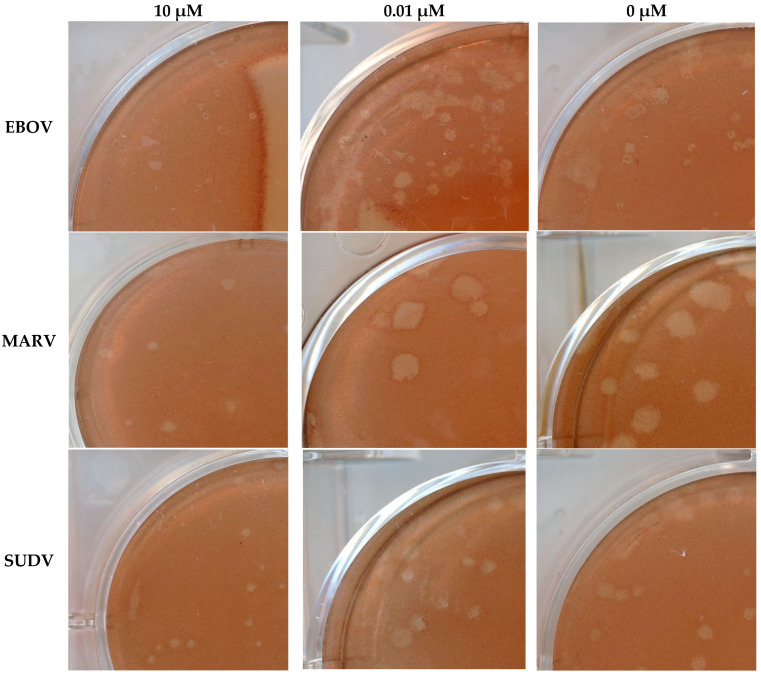
Representative images of virus plaques at different concentrations of probenecid. All plates were scanned at 800 dpi, with four plates per scan. All images above were captured at 80% zoom and aspect ratios/sizes maintained.

Probenecid treatment at higher concentrations, i.e., 10 µM and 1 µM, resulted in reduced plaque sizes compared to those in the two lowest concentrations and control wells for all three viruses (Table 2 and Figure 2). For EBOV, wells overlaid with the two highest drug concentrations contained small to very small plaques. In comparison, wells overlaid with the two lower concentrations or the control article contained a mixture of small/medium plaques (consistent with expected plaque morphology). For MARV, wells overlaid with the two highest drug concentrations contained medium plaques. In comparison, wells overlaid with the two lower concentrations or the control article contained a mixture of medium- to large plaques (consistent with the expected plaque morphology). For SUDV, wells overlaid with the two highest drug concentrations contained small plaques. In comparison, wells overlaid with the two lower concentrations or the control article contained medium plaques (consistent with expected plaque morphology).

These results show that probenecid inhibits filovirus replication in vitro. Probenecid resulted in reduced plaque counts and smaller plaque sizes compared to the control treatment. Future experiments in an appropriate animal model would be necessary to determine whether the efficacy observed in Vero E6 cells translates to in vivo efficacy. Still, the host-directed mechanism of action suggests probenecid could be a promising candidate for in vivo efficacy against filoviruses.

## 4. Discussion

Future filovirus therapy will likely focus on small-molecule inhibitors targeting key steps in the viral life cycle, such as attachment to host cells, membrane fusion, and viral RNA synthesis. Current research is exploring pan-filovirus inhibitors that could target multiple filoviruses, including EBOV and MARV, to address the urgent need for new drugs [19]. A deeper understanding of the molecular mechanisms underlying viral replication may facilitate the development of host-directed anti-filovirus agents. In this study, we examined a safe and readily available drug that has been shown to have pan-respiratory-virus effectiveness and to reduce pro-inflammatory activity. This study demonstrated that probenecid suppressed replication of EBOV, SUDV, and MARV in Vero E6 cells, reducing plaque numbers and sizes in a concentration-dependent manner. Potency was greatest against EBOV and modestly lower for MARV and SUDV under these conditions. At the same time, smaller plaques across the 10–1 μM range suggested drug effects limiting cell spread and replication.

The results are consistent with previous reports showing the efficacy of probenecid across several respiratory RNA viruses, including strains and variants of SARS-CoV-2, influenza A (including H5N1 and H7N9), RSV, and HMPV, both in vitro, in vivo, and in the clinic [10,11,12,13,14,16]. Antiviral activity across a range of viruses is possible, as probenecid can impact conserved host pathways, including MAPK signaling [8,9,10,15]. Notably, the reduction in plaque size also supports an effect on virus spread linked to virus replication.

The results from this study support the utility of probenecid as a treatment against these filoviruses. Future clinical trials may evaluate the efficacy of probenecid as a single agent or in combination with other agents to enhance antiviral efficacy and resilience. Combining a host-directed antiviral with a direct-acting antiviral could produce additive or synergistic effects, help to further mitigate resistance, and enable dose-sparing. For filoviruses, nucleoside/nucleotide polymerase inhibitors, such as remdesivir and the next-generation analog obeldesivir, are potential combination agents because they directly inhibit the viral RNA-dependent RNA polymerase [20,21,22].

## Figures and Tables

**Table 1:** The percent in vitro plaque reduction following probenecid treatment.

[Probenecid]	1000 µM	100 µM	10 µM	1 µM	0.1 µM	0.01 µM	IC_50_ [µM]
EBOV	100%	80%	60%	44%	24%	0%	3
MARV	100%	60%	41%	41%	32%	0%	8
SUDV	100%	65%	44%	31%	6%	0%	13

**Table 2:** Relative plaque sizes at different probenecid concentrations.

Probenecid	10 µM	1 µM	0.1 µM	0.01 µM	0 µM
EBOV	very small	very small	small/medium	small/medium	small/medium
MARV	medium	medium	medium/large	medium/large	medium/large
SUDV	small	small	medium	medium	medium

## Data Availability

All the data are contained within the manuscript. The data supporting the reported results are available in archived datasets generated at Texas Biomed in San Antonio, TX.
